# A Robust Feedforward Model of the Olfactory System

**DOI:** 10.1371/journal.pcbi.1004850

**Published:** 2016-04-11

**Authors:** Yilun Zhang, Tatyana O. Sharpee

**Affiliations:** 1 Computational Neurobiology Laboratory, The Salk Institute for Biological Studies, La Jolla, California, United States of America; 2 Department of Physics, University of California San Diego, La Jolla, California, United States of America; Université Paris Descartes, Centre National de la Recherche Scientifique, FRANCE

## Abstract

Most natural odors have sparse molecular composition. This makes the principles of compressed sensing potentially relevant to the structure of the olfactory code. Yet, the largely feedforward organization of the olfactory system precludes reconstruction using standard compressed sensing algorithms. To resolve this problem, recent theoretical work has shown that signal reconstruction could take place as a result of a low dimensional dynamical system converging to one of its attractor states. However, the dynamical aspects of optimization slowed down odor recognition and were also found to be susceptible to noise. Here we describe a feedforward model of the olfactory system that achieves both strong compression and fast reconstruction that is also robust to noise. A key feature of the proposed model is a specific relationship between how odors are represented at the glomeruli stage, which corresponds to a compression, and the connections from glomeruli to third-order neurons (neurons in the olfactory cortex of vertebrates or Kenyon cells in the mushroom body of insects), which in the model corresponds to reconstruction. We show that should this specific relationship hold true, the reconstruction will be both fast and robust to noise, and in particular to the false activation of glomeruli. The predicted connectivity rate from glomeruli to third-order neurons can be tested experimentally.

## Author Summary

Many olfactory systems are capable of accurately sensing a minimum of thousands of different odorants using as few as hundreds of different receptors. This compression raises the possibility that the mathematical properties of compressed sensing might be relevant to olfaction, similar to how these properties were found relevant to other sensory systems. In olfaction, previous applications of compressed sensing algorithms relied on the dynamics of neural circuits to reconstruct high dimensional signals. Such approaches are relatively temporally inefficient and sensitive to noise. To overcome these problems, we propose a purely feedforward compressed sensing model of the olfactory system where high dimensional signals can be recovered with a single feedforward layer of neural processing. The reconstructions are shown to be robust to noise, account for a number of experimental observations, and because of the feedforward structure are temporally efficient. Using the model, we make predictions that can be tested in future experiments with respect to optimal connectivity within the olfactory system. Our results indicate that feedforward neural architectures can provide an efficient way to implement compressed sensing in neural systems.

## Introduction

Although it is still debated how many different odorants humans can perceive, the most commonly cited number is on the order of 10^4^ [1–3], much greater than the 500 olfactory receptor neuron (ORNs) types. Many other species, including both vertebrates and insects, have the same order of magnitude of ORN types or even fewer (around 1000 in mice, 50 in Drosophila). The order of magnitude difference between the number of odorants and ORN types implies that humans as well as other species rely on compressed representations, potentially following the principles of compressed sensing [4–7].

In the compressed sensing framework [4], sparse high dimensional signals can be accurately reconstructed using a small number of measurements provided that the input signals are sparse. Natural odors are sparse in the sense that they are dominated by a few molecular components [8–10]. The relevance of compressed sensing algorithms to olfactory coding is reinforced by the anatomical organization of the olfactory system. High dimensional odor signals are compressed into a low-dimensional representation in terms of the activity of a relatively small number of glomeruli in the olfactory bulb, in the case of vertebrates, or the antennal lobe in the case of invertebrates. The standard compressed sensing algorithm performs signal reconstruction as a constrained *ℓ*_1_ minimization [4]. Such optimization can be solved through neural dynamics [5, 6], but the resulting reconstructions were considerably less fault tolerant than observed experimentally. For example, mice olfactory discrimination remains essentially intact when half of glomeruli are disabled [11] whereas theoretical reconstructions fail at this level of signal interference [5]. Furthermore, signal reconstruction based on dynamical optimization by construction requires more time for signal recognition compared to feedforward reconstruction schemes. Here we describe a feedforward reconstruction scheme based on compressed sensing ideas that is both fault tolerant and matches the main features of the organization of the olfactory system. The results demonstrate that a purely feedforward network is capable of robustly compressing/decompressing binary signal without dynamical optimization.

## Models and Methods

### A compressed sensing model of the olfactory system

We begin by reviewing the main results from compressed sensing literature as they pertain to olfactory coding. The odor signal *s*^0^ can be described as a binary vector of length *N* where each element is either 1 or 0 depending upon whether a given molecular component is present or not in the odor. We refer to the number *K* of nonzero components in the odor as the odor sparsity. The main premise of compressed sensing is that a sparse signal *s*^0^ can be compressed into a vector *x* = *As*^0^ of length *M* < *N* and then recovered with high reconstruction quality provided *K* ≪ *N*. The encoding matrix *A* has dimensions *M* × *N*; its matrix elements can be chosen randomly. With this setup, the original signal *s*^0^ can be recovered exactly from the convex *ℓ*_1_ optimization problem [4]

$$\begin{array}{c} \hat{s}={\arg\min||s||}_{1}\;\;\;\;\;\text{subject}\;\text{to}\;x=As^{0}. \end{array}$$ (1)

Although the *ℓ*_1_ minimization problem can be solved in polynomial time, it is not straightforward to implement such optimization algorithms in a neural circuit. One solution involves a two-layer neural network that perform similar *ℓ*_1_ minimization through neural dynamics [6]. However, this imposes certain requirements on the structure of recurrent connections in the second layer together with a static nonlinear activation function. Another alternative implementation relies on *ℓ*_2_ minimization instead of *ℓ*_1_. In this case, the reconstruction is obtained simply as $\hat{s}={\left(A^{T}A\right)}^{-1}A^{T}x$ where the ^−1^ represents a pseudo-inverse relation. However, such an approach does not produce exact signal reconstruction [7] and would predict much larger errors than observed in olfactory experiments.

### Robust feedforward reconstruction of sparse odors

We now propose a model for the olfactory system, which can compress and robustly recover sparse binary signal with high probability, without using any dynamical optimization. The solution is based on a nonlinear binary encoding model instead of the linear encoding model used in the conventional compressed sensing approach. Specifically, the compressed vector *x* has the form of a threshold function $x_{i}=H\left(x_{i}^{l}-\theta_{c}\right)$ where *x*^*l*^ = *As*^0^ and H is the Heaviside step function with H(0)=1. We assume that the measurement matrix (affinity matrix) *A* is a *M*×*N* random binary matrix where each element is chosen independently to be either 1 or 0 with equal probability *p* and 1 − *p*, respectively. It is worth mentioning that while we use a random connectivity matrix in our model, we do not assume that this matrix differs across individuals. Rather, the randomness is meant to characterize how well the system works in the absence of specificity between odorants and glomeruli identity. By extending the definition of H to vectors, the measurement vector *x* can be compactly written as

$$\begin{array}{c} x=H(As^{0}-\theta_{c}), \end{array}$$ (2)

where *θ*_*c*_ = 1, reflects that all measurements larger than 1 are set to 1 so that *x* is binary. This corresponds to a binary model of glomeruli activity described by the binary vector *x*. The threshold value of *θ*_*c*_ = 1 corresponds to a logical OR operation, so that glomerulus *k* will be activated if any of the odor components that are associated with inputs to this glomerulus are activated.

To reconstruct the original signal, the glomeruli activity *x* are projected to another layer of neurons (neurons in the olfactory cortex of vertebrates or Kenyon cells in the mushroom body of insects) which has the same dimension as the original signal *s*^0^. The activity of neurons in this layer is denoted by vector $\hat{s}$ which has the same dimensionality *N* as the original signal *s*^0^. The reconstructed signal can be computed as

$$\begin{array}{c} \hat{s}=H\left(W^{T}x-\theta_{r}\right), \end{array}$$ (3)

where *θ*_*r*_ is the activation threshold for neurons in the reconstruction layer. The reconstruction matrix *W* equals the measurement matrix *A* normalized to 1 by column, i.e. *W*_*ki*_ = *A*_*ki*_/∑_*k*_ *A*_*ki*_. With this normalization, the reconstruction threshold *θ*_*r*_ = 1 corresponds to logical AND operation. That is, odor component *i* will be detected as present if all glomeruli that feed signals to node *i* in the reconstruction layer are activated. Below we will present most of the results for *θ*_*r*_ = 1 and then analyze how the reconstruction quality and recovery robustness depend on this threshold. We will also determine the optimal connectivity ratio from the compression to the reconstruction layer that maximizes the fidelity of reconstructions.

### Maximal information transmission

Our feedforward model can be thought of as an information transmission channel that compresses, transmits, and decompresses a sparse binary signal. To find the optimal network configuration, we seek to maximize mutual information between the input and output of the channel as has been done to characterize performance in the visual and other sensory systems. The mutual information between *s*^0^ and $\hat{s}$ is given by

$$\begin{array}{c} I\left(s^{0},\hat{s}\right)=\sum_{s^{0}}\sum_{\hat{s}}P\left(\hat{s}|s^{0}\right)P\left(s^{0}\right)\log_{2}\frac{P(\hat{s}|s^{0})}{P(\hat{s})}. \end{array}$$ (4)

For a given signal sparsity *K*, the conditional probability $P(\hat{s}|s^{0})$ of the reconstructed signal $\hat{s}$ given the original signal *s*^0^ can be computed as:

$$\begin{array}{c} P\left(\hat{s}|s^{0}\right)=p_{\text{false}}^{N_{err}}{\left(1-p_{\text{false}}\right)}^{\left(N-K-N_{err}\right)}, \end{array}$$ (5)

where $p_{\text{false}}\equiv P\left({\hat{s}}_{i}=1\;|\;s_{i}^{0}=0\right)$ is the probability of false detection for an odor component and $N_{err}=\left|\right|\hat{s}{\left|\right|}_{0}-K$ is the number of false detection events for the odor *s*^0^. We note that for *θ*_*r*_ = 1, the probability to miss an odor component is zero provided this odor component activates at least one of the glomeruli. In this regime, the information is fully determined by the false detection rate *p*_false_, and as we show below decreases proportionally with *p*_false_.

Assuming a uniform prior over individual odor components $P(s^{0})=1/\left(\begin{array}{c} N\\ K \end{array}\right)$, one can also compute the probability distribution of reconstructed signals:

$$P(\hat{s})=\sum_{s^{0}}P(\hat{s}|s^{0})P(s^{0})=\frac{\left(\begin{array}{c} K+N_{err}\\ K \end{array}\right)}{\left(\begin{array}{c} N\\ K \end{array}\right)}p_{\text{false}}^{N_{err}}{\left(1-p_{\text{false}}\right)}^{\left(N-K-N_{err}\right)}$$ (6)

Putting together Eqs (4)–(6), the mutual information can be written as

$$I(s^{0},\hat{s})\;={\text{log}}_{2}\left(\begin{array}{c} N\\ K \end{array}\right)-\sum_{N_{err}=0}^{N-K}\left(\begin{array}{c} N-K\\ N_{err} \end{array}\right)p_{\text{false}}^{N_{err}}{\left(1-p_{\text{false}}\right)}^{\left(N-K-N_{err}\right)}{\text{log}}_{2}\left(\begin{array}{c} K+N_{err}\\ K \end{array}\right).$$

When (*N* − *K*)*p*_false_ ≪ 1, the summation above can be well approximated by its leading nonzero term

$$\begin{array}{l} \sum_{N_{err}=0}^{N-K}\left(\begin{array}{c} N-K\\ N_{err} \end{array}\right)p_{\text{false}}^{N_{err}}{\left(1-p_{\text{false}}\right)}^{\left(N-K-N_{err}\right)}{\text{log}}_{2}\left(\begin{array}{c} K+N_{err}\\ K \end{array}\right)\\ \approx (N-K)p_{\text{false}}{\text{log}}_{2}(K+1), \end{array}$$ (7)

so that the expression for the mutual information becomes:

$$I(s^{0},\hat{s})\approx {\text{log}}_{2}\left(\begin{array}{c} N\\ K \end{array}\right)-(N-K)p_{\text{false}}{\text{log}}_{2}(K+1).$$ (8)

Thus, for given *N* and *K*, maximizing $I(s^{0},\hat{s})$ can be approximated by minimizing the probability of false detection *p*_false_.

## Results

### Optimal connectivity rate

The false detection rate that appears in Eq 8 can be computed as

$$\begin{array}{c} \begin{array}{cc} p_{\text{false}} & \equiv P({\hat{s}}_{i}=1\;|\;s_{i}^{0}=0)\\ & =\sum_{k=1}^{M}P({\hat{s}}_{i}=1\;|\;||T_{i}{\left|\right|}_{0}=k)P(||T_{i}{\left|\right|}_{0}=k\;\left|\;s_{i}^{0}=0\right)\\ & =\sum_{k=1}^{M}{\left[1-{\left(1-p\right)}^{K}\right]}^{k}\frac{\left(\begin{array}{c} M\\ k \end{array}\right)p^{k}{\left(1-p\right)}^{M-k}}{1-{\left(1-p\right)}^{M}}\\ & =\frac{1}{1-{\left(1-p\right)}^{M}}\sum_{k=0}^{M}{\left[1-{\left(1-p\right)}^{K}\right]}^{k}\left(\begin{array}{c} M\\ k \end{array}\right)p^{k}{\left(1-p\right)}^{M-k}-\frac{{\left(1-p\right)}^{M}}{1-{\left(1-p\right)}^{M}}\\ & =\frac{{\left[1-p{\left(1-p\right)}^{K}\right]}^{M}-{\left(1-p\right)}^{M}}{1-{\left(1-p\right)}^{M}}, \end{array} \end{array}$$ (9)

where *T*_*i*_ ≡ {*x*_*k*_ ∈ *x*|*A*_*ki*_ = 1}, and *p* is the average connectivity rate from the compression to the reconstruction layer. In the last line above we use the binomial expansion. Because we are interested in the regime where *M* is large, we have (1 − *p*)^*M*^ ≪ [1 − *p*(1 − *p*)^*K*^]^*M*^ ≪ 1 as long as *p* is not too small. Thus, Eq 9 can be approximated with great accuracy by the following simple equation:

$$\begin{array}{c} p_{\text{false}}={\left[1-p{\left(1-p\right)}^{K}\right]}^{M}. \end{array}$$ (10)

**Fig 1:**
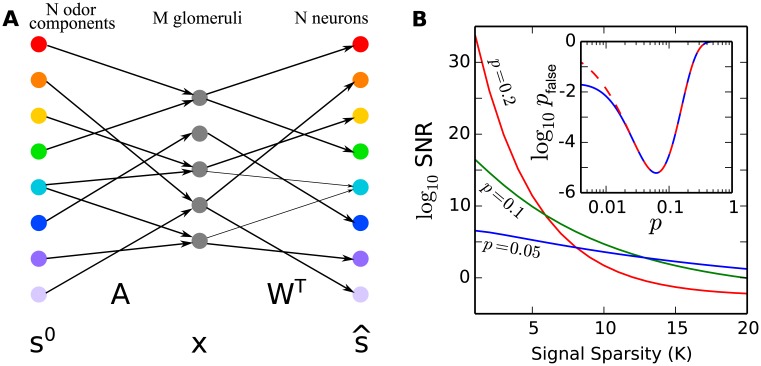
**(A) Illustration of the model structure.** An odor is represented by a sparse binary vector *s*^0^ of its mono-molecular components. This signal is compressed into the activities of *M* glomeruli represented by a binary vector *x* through a binary measurement matrix *A*. The signal is then recovered as the activities of *N* neurons in the mushroom body or olfactory cortex represented by a binary vector $\hat{s}$ through another matrix *W*^*T*^. (B) The signal-to-noise ratio (SNR) as a function of signal sparsity *K* where *N* = 10000 and *M* = 500. For a given *K*, there is a optimal connectivity rate *p* = *p*_*m*_ that maximizes SNR. At the same time, even for a system optimized to a given *K*, decreasing *K* still increases SNR. Inset: false detection rate *p*_false_ as a function of average connectivity *p*; *M* = 500 and *K* = 15 are chosen for this illustration. Solid line is exact formula, while dashed line is the approximation using Eq 10. We can see that Eq 10 is a very good approximation to the exact formula when *p* is not too small.

As shown in the inset of Fig 1B, Eq 10 provides an accurate approximation when the connectivity *p* is not too sparse. Since our main interest is near the optimal connectivity rate (see below) where Eq 10 is very accurate, we will use Eq 10 unless specified.

As expected, the false detection rate *p*_false_ decreases as the number of glomeruli *M* increases and as the signal sparseness *K* decreases. Importantly, for a given *M* and *K*, there is an optimal *p*, which we refer to as *p*_*m*_, that minimizes *p*_false_, as shown in Fig 1B. Taking ∂*p*_false_/∂*p* = 0 leads to

$$\begin{array}{c} p_{m}=\frac{1}{K+1}. \end{array}$$ (11)

It is worth noticing that the optimal connectivity *p*_*m*_ is independent of the number of glomeruli *M*, and depends only on the signal sparseness *K*. Thus, optimal connectivity depends exclusively on the level of sparseness of signals in the environment and can be determined prior to any measurements on neural circuits.

For an optimal connectivity *p* = *p*_*m*_, the probability of fault activation decreases exponentially as *M* increases and thus can be very small. This indicates that the proposed feedforward compression-reconstruction scheme from Fig 1A can achieve exact recovery with high probability.

**Fig 2:**
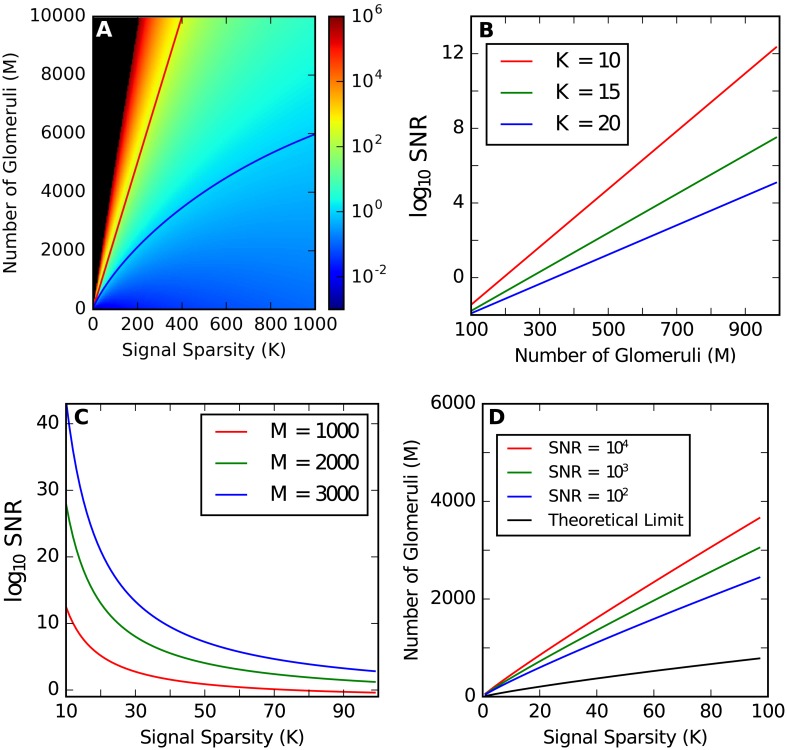
Signal-To-Noise-Ratio (SNR) of the recovered signal in our model. *N* = 10000 is used. (A) SNR as a function of *K* and *M*. Black is shown for SNR > 10^6^. The blue line shows SNR = 1, and the red line shows SNR = *K*, i.e. one error occurs on average. (B) Optimal SNR as a function of *M*. (C) Optimal SNR as a function of *K*. (D) Number of glomeruli required to reach threshold SNR when optimal connectivity rate is used.

To test the reconstruction quality, we compute the signal-to-noise-ratio (SNR) of the recovered signal. Since all nonzero components in the original will be recovered, the only source of errors in the reconstructed signal are due to false detection rates. Therefore, we can define the SNR of recovered signal as

$$\begin{array}{c} \text{SNR}=\frac{\left|\right|s^{0}{\left|\right|}_{0}}{<||\hat{s}{\left|\right|}_{0}>-\left|\right|s^{0}{\left|\right|}_{0}}=\frac{K}{\left(N-K\right)p_{\text{false}}}, \end{array}$$ (12)

as shown in Fig 2A–2C, where < ⋅ > denotes the expectation value. We can see from Fig 2B that the SNR increases exponentially with *M*. For our case where *K* ≪ *N*, we can achieve a high SNR for a number of glomeruli *M* much smaller than the number of odor components *N* or, equivalently, the number of third-order neurons.

### Compression rate and sparsity

A key characteristic of a compression algorithm is the compression ratio *α* ≡ *M*/*N*. In previous compressed sensing frameworks, the critical compression ratio *α*_*c*_ above which the signal can be perfectly recovered was shown to only depend on the relative signal sparsity *f* ≡ *K*/*N*. As *f* → 0, *α*_*c*_(*f*)∼ −*f* log *f* [12]. To compute the critical compression ratio for our reconstruction algorithm, we note that from Eq 12, log *p*_false_ = log *f* − log(1 − *f*) − log SNR. In the strong compression limit where *f* ≡ *K*/*N* is small, this yields

$$\begin{array}{c} \log p_{\text{false}}\approx \log f-\log\text{SNR}. \end{array}$$ (13)

On the other hand, for the optimal connectivity rate *p*_*m*_ and large *K*, log *p*_false_ can also be simplified using Eq 10 as follows:

$$\begin{array}{c} \begin{array}{cc} \log p_{\text{false}} & =M\log\left[1-\frac{1}{K+1}{\left(1+\frac{1}{K}\right)}^{-K}\right]\\ & \approx M\log\left(1-\frac{1}{K+1}e^{-1}\right)\approx -\frac{M}{eK}=-\frac{\alpha_{\text{SNR}}}{ef}. \end{array} \end{array}$$ (14)

where *α*_SNR_ is defined as the compression rate to achieve a certain SNR. Combining Eqs 13 and 14, in the limit of strong compression where *f* → 0, the critical compression ratio behaves as *α*_SNR_ ∼ −*f* log *f*. We note that care should be taken when the SNR becomes comparable to or larger than *N* because 1/*f* = *N*/*K* ≤ *N*, so that log SNR cannot be neglected when *f* → 0.

The obtained critical compression rate can be compared to its theoretical limit. The latter corresponds to the minimal number of bits required to encode a sparse signal:

$$M_{min}=\left\lceil {\text{log}}_{2}\left(\begin{array}{c} N\\ K \end{array}\right)\right\rceil ,$$ (15)

where ⌈*x*⌉ is the smallest integer not less than *x*. When *N* and *K* are large but *f* ≡ *K*/*N* is small, using Stirling’s approximation, we obtain that

$$\begin{array}{c} \begin{array}{cc} M_{min}\times \log2 & \approx N\log N-K\log K-(N-K)\log(N-K)\\ & \approx K\log N-K\log K+K=K-K\log f, \end{array} \end{array}$$ (16)

This yields that the theoretically possible compression ratio *α*_*min*_ in the strong compression limit of *f* → 0 as

$$\begin{array}{c} \alpha_{min}\to f\log_{2}e/f, \end{array}$$ (17)

which also yields *α*_*min*_ ∼ −*f* log *f* as *f* → 0.

Notice that although both *α*_SNR_ and *α*_*min*_ behave as −*f* log *f* for *f* → 0, they have different proportionality coefficients. To be more specific, *α*_SNR_ ∼ *ef* log 1/*f* while *α*_*min*_ ∼ (log 2)^−1^ *f* log 1/*f*. As a result, *α*_SNR_/*α*_*min*_ → *e* log 2 ≈ 1.88 as *f* → 0. Thus, the number of glomeruli needed in our model is about twice the theoretical limit but is achieved here with an extremely simple feedforward encoding model.

As shown in Fig 2D, the number of required glomeruli increases sub-linearly with *K*, and logarithmically with SNR. In practice, with only a few times more glomeruli than the theoretical limit, a very high SNR can be achieved.

### Robustness and fault tolerance

Advances in experimental techniques provide opportunities to test our theory under the circumstances of extreme genetic manipulations. For example, following a genetic manipulation that caused most olfactory receptor neurons to express a single odorant receptor M71, the M71 ligand acetophenone activates half of the glomeruli. Despite this drastic manipulation, mice can still readily detect other odors in the presence of acetophenone, while their discrimination performance is only moderately compromised [11]. This result is consistent with our model. Assume there are *M* glomeruli in our model and half of them are always turned on (corrupted). Such a system is equivalent to a model with only *M*/2 glomeruli, since the anomalously activated glomeruli will not affect signal recovery. Thus, the odor signal can still be recovered, but the SNR is decreased, which is in agreement with the experimental result. As a comparison, in previous compressed sensing framework, one can only allow a small percentage of corrupted glomeruli even when *M* > *N* [4].

In another set of experimental studies, part of the glomeruli in mice are removed or disabled [13–15]. It is shown that the ability to discriminate odors and simple odor mixtures is not impaired even when most of the glomeruli are removed or disabled. This seemingly surprising finding is also consistent with our model. From previous results, one can see that decreasing *M* will only lead to larger noise in the recovered odor signal but not to a failure of the system if the activation threshold for neurons in the reconstruction layer can be properly adapted to the new *M*. Assume the mice need SNR > *ν* to discriminate odors. When *K* is small, the minimal *M* needed for discrimination is

$$\begin{array}{c} M_{low}=\frac{\log\frac{K}{N\nu }}{\log[1-p{\left(1-p\right)}^{K}]}. \end{array}$$ (18)

From experiment data, *p* ≈ 0.05 (although this is a very rough estimation, see [11, 16–18]). One can check that the equation above is insensitive to variations in *K* and *Nν* over a broad range. If we assume *K* < 10 (as in the experiments) and *Nν* is within the range of 10^4^ ∼ 10^5^, then *M*_*low*_ is roughly between 200 and 300, or around 20% of the glomeruli, which is in good agreement with the data in those experiments.

**Fig 3:**
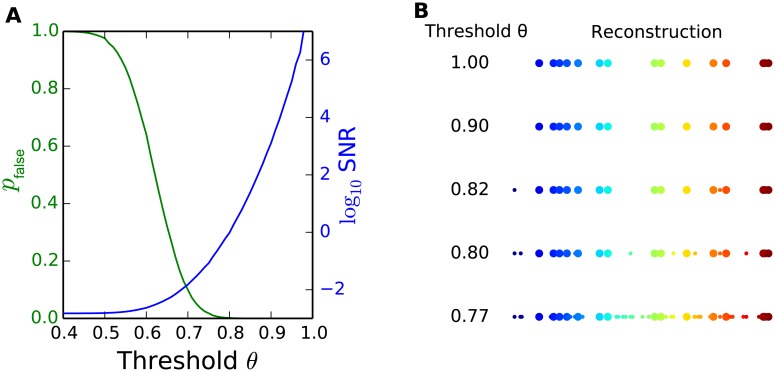
Demonstration of the accuracy-robustness trade-off. *N* = 10000, *K* = 15, *M* = 1000 and the optimal connectivity rate are used. (A) *p*_false_ and SNR for different activation thresholds at the reconstruction stage. With lower recovery thresholds, the robustness of the system to recovery noise increases, while the false detection rate increases, and the SNR of recovered signal decreases. (B) An example of the recovered signal with different recovery thresholds. True signal is shown in big colored dots, while the reconstruction error is represented by small colored dots. As we lower the threshold, the recovered signal becomes noisier.

On the other hand, our model can tolerate negative gloleruli noise (false negative) by changing its recovery threshold *θ*_*r*_. Although we use *θ*_*r*_ = 1 in our results for analytical solution, it is very likely that real biological systems would use a lower threshold *θ*_*r*_. With *θ*_*r*_ < 1, the SNR is somewhat lower, as shown in Fig 3, yet the system is more robust to noise in the reconstruction stage since the activation of a third-order neuron doesn’t require all of its connected gloleruli to be active and it also leaves room for odor generalization and pattern completion [19]. Indeed, when the threshold at the reconstruction stage is less than 1, the reconstruction can tolerate some incompleteness in the glomeruli activation patterns. Real biological systems likely have the ability to adaptively change the activation threshold in order to balance the needs of high quality reconstruction and pattern completion.

Our model is shown to be very robust and fault tolerant, and this robustness is achieved with accuracy. As one can see, each glomerulus in the model only contains part of the information about the original signal. Because the measurement matrix *A* is random, no single glomerulus or cluster contains more or unique information, so any subset of the glomeruli could recover the original signal. The more glomeruli there are, the better recovery quality (SNR) can be achieved. Thus, removing or disabling part of the glomeruli will not change the system qualitatively, but will make the recovered signal more noisy, up to a point where noise becomes comparable to the true signal at which point the reconstruction fails. For a real biological system, it is reasonable to assume that the recovered signal has very high SNR, which also means high redundancy, as is observed experimentally.

## Discussion

### Predicted optimal connectivity rate compared with experimental data

From our analysis we observed that for a given level of signal sparseness *K*, there is an optimal connectivity rate *p*_*m*_ that maximizes SNR as well as the mutual information. Assuming that the biological system is adapted to a given value of odor sparseness in its environmental niche, one can essentially make predictions on the connectivity rate of matrix *A*. This is followed by another prediction that the percentage of glomeruli activated by a single odorant should be close to the percentage of glomeruli that could activate a neuron in olfactory cortex or a Kenyon cell, and this number should be similar among species which operate in similar olfactory environments. The latter prediction should be easier to test, since the number of coexisting odorants in the environment is hard to measure.

Fortunately, previous experiments have gathered sufficient data to test our prediction indirectly. It has been shown that in Drosophila, 9% of the glomeruli have a strong response to an odorant [20], while the connectivity rate between glomeruli and Kenyon Cells is 6.5% [21] to 12.5% [22]. (The latter number is obtained based on the average number of claws per Kenyon cell measured in [22]) These estimates are consistent with model predictions. Furthermore, in the locust, a typical projection neuron responds to about half of the odorants [23], while the connectivity rate between projection neurons and Kenyon Cell is also around 50% [24], which is also consistent with our prediction.

We can see that the connectivity rate is very different between species. Such differences can be unified in our model as the adaptation to different environmental niches. The locust has an anomalously high connectivity rate (50%), which in our model implies that its olfactory system is adapted to extreme odor sparseness tuned to odors with primarily a single component (*p*_*m*_ = 0.5 when *K* = 1). Similarly, Drosophila is adapted to sense odors composed of a mixture of about 10 odor components, while mice are tuned to detect a mixture of about 20 mono-molecular odors. In general, our model predicts that species with sparse connectivity will behave better in environments with complex odor mixtures, while species with dense connectivity have better performance in detecting simple odor mixtures.

### Structural and functional evidence

In addition to the predictions above, further experimental evidence supports the structure of our model, in particular the approximate logical OR/AND operations associated with the compression/reconstruction stages, respectively. For example, it has been observed experimentally that Kenyon Cells in Drosophila receive convergent input from different glomeruli and require several inputs to be co-active to spike [25]. This is consistent with our threshold activation function which at the reconstruction stage uses a logical AND operation.

Functionally, experiments have shown that locust Kenyon cells are individually much better than projection neurons from glomeruli at detecting a single odorant; Kenyon cells that respond to an odorant also often respond to odor mixtures containing it [26]. This observation agrees with our assumption that each Kenyon cell only responds to one odorant and it will respond when an odor mixture contains that odorant.

### Stereotyped versus non-stereotyped connectivity

Since the affinity matrix *A* is determined genetically, all the connections in our model are predetermined before birth. There is some debate about such stereotypy versus random connectivity, and a compressed sensing model of olfaction based on random connections from glomeruli to mushroom body has been proposed [27]. Yet, our model supports both stereotyped and non-stereotyped projection from glomeruli to the mushroom body/olfactory cortex because the model is invariant under the exchange of neurons within the same layer. In order to verify such predetermination, one needs to obtain a detailed connectivity map from glomeruli to the mushroom body/olfactory cortex for different individuals, which is experimentally very challenging. An indirect approach to verify the predetermined connectivity hypothesis could be through an examination of innate behaviors that should depend primarily on predetermined connections. If one could relate innate behaviors to projections between glomeruli and the mushroom body/olfactory cortex, it would then provide additional supporting evidence for the genetically predetermined structural connectivity of the feedforward model.

### Effective feedforward model for non-feedforward structure

The feedforward structure of our model is an effective approximation to the more complicated structure of biological olfactory system where recurrent and feedforward-feedback connections exist. For example, it has been observed that inhibitory interneurons modulate neuronal responses in the olfactory bulb [28, 29]. In linear dynamic systems, such feedforward-feedback structure could be mathematically modeled as a pure feedforward system with different effective feedforward connectivity. Suppose that we add a layer of interneurons *z* in Fig 1 that is connected to the glomeruli layer *x* by feedforward-feedback connectivity *B*. Then the linear dynamics of the system are $\dot{x}=-x+As^{0}-B^{T}z$ and $\dot{z}=-z+Bx$, where we assume *B* is feedforward excitatory and feedback inhibitory. The steady state solution is *x* = (*I* + *B*^*T*^ *B*)^−1^ *As*^0^, which is the same for a pure feedforward system, except that connectivity *A* is replaced by (*I* + *B*^*T*^ *B*)^−1^ *A*. This analysis is not exact if the activation function is nonlinear. In general, the feedforward-feedback system in steady state with a nonlinear activation function does not have an equivalent feedforward system, but one can still write the linear perturbation when neurons receive only weak inputs, which allows a feedforward approximation. Such a feedforward approximation is supported by experimental observations that the representations of odor mixtures in mouse glomeruli can be explained well by the summation of the glomeruli responses to their components [30].

One advantange of the effective feedforward model is that it enables an adaptive affinity matrix even with pre-determined connectivity. In the feedforward-feedback architecture mentioned above, the effective affinity matrix is (*I* + *B*^*T*^ *B*)^−1^ *A*, where *A* is the pre-determined affinity matrix encoded in the genes, while *B* could be a learned matrix adapted to the environment. From this perspective, the existence of interneurons in both insects and vertebrates [31, 32], as well as adult neurogenesis in the olfactory bulb of mammals [33], could play the role of adjusting the effective affinity matrix for the purpose of adaptation.

### Comparison with *ℓ*_1_ minimization algorithm

**Fig 4:**
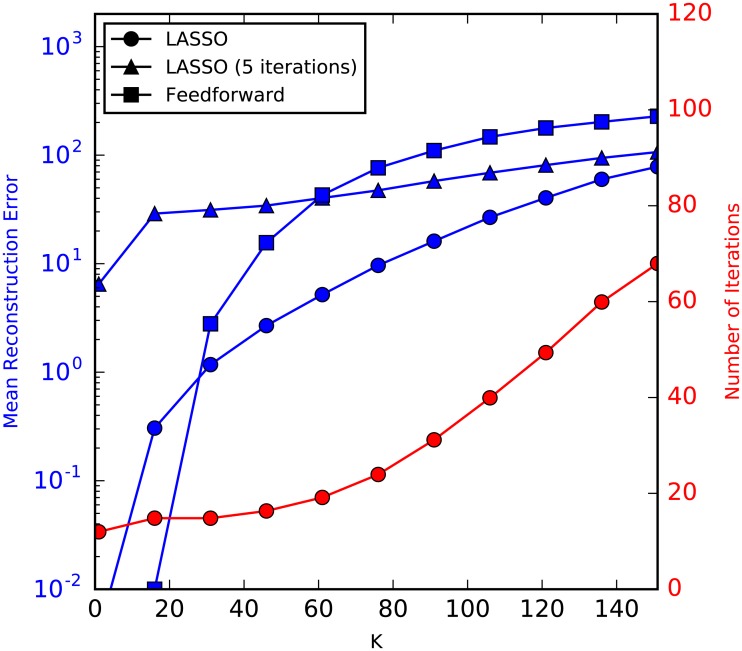
Comparison of the performance of feedforward architecture with that of LASSO. For this example, we chose *N* = 1000 and *M* = 500. Linear measurement is used for LASSO. Feedforward architecture performs well when the signal is very sparse, while LASSO has lower reconstruction error as *K* increases, at the price of increasingly more iterations. On the other hand, if we constrain the number of iterations, LASSO still performs better when *K* is large, but significantly worse with very sparse signals.

We compare the performance of our feedforward architecture with the often-used LASSO *ℓ*_1_ minimization algorithm [34] provided by the Python scikit-learn library

$$\begin{array}{c} \min_{\hat{s}}\frac{1}{2M}||A\hat{s}-{x||}_{2}^{2}+\beta ||\hat{s}{\left|\right|}_{1}, \end{array}$$ (19)

where *N* = 1000, *M* = 500, *β* = 0.001 are used. Linear measurement *x* = *As*^0^ is used for LASSO. For each *K*, we conduct 100 experiments with different random measurement matrices and signals, and compute the average of the reconstruction errors $\left|\right|\hat{s}-s^{0}{\left|\right|}_{1}$ as well as the number of iterations used in LASSO. We also compute the mean reconstruction error when only 5 iterations are used in LASSO as a comparison. The results are shown in Fig 4. As shown in the figure, the feedforward architecture has a lower reconstruction error when the signal is very sparse, while LASSO has a lower reconstruction error than the feedforward architecture when *K* becomes larger. However, the number of iterations also increases as the signal becomes denser. If we restrict the number of iterations to 5 in the LASSO (equivalent to setting a maximum response time), LASSO performs much worse when the signal is very sparse. But as *K* increases, it still has a lower reconstruction error than the feedforward architecture.

### Performance with non-sparse signal

One drawback of this feedforward architecture is that it may not be able to achieve both compression and high-quality reconstruction simultaneously when the signal is not sparse. Unlike the *ℓ*_1_ minimization method where the number of measurements required to reconstruct the signal will never exceed signal length *N* (*N*/2 for binary signal)[35, 36], the feedforward architecture may need more measurements than the signal length to accurately reconstruct the signal. This can be seen by restoring the term in Eq 13 that we have previously neglected assuming that *f* is small

$$\begin{array}{c} \log p_{\text{false}}=\log f-\log\left(1-f\right)-\log\text{SNR}. \end{array}$$ (20)

Combining this with Eq 14 that remains the same when *f* is not small, we obtain:

$$\begin{array}{c} \alpha_{\text{SNR}}=ef\log\text{SNR}+ef\log\left(f^{-1}-1\right), \end{array}$$ (21)

which could be larger than 1 when *f* is not small. Thus, the feedforward computation may require number of measurements that are larger than the input dimensionality to achieve reliable reconstruction.

From another perspective, we can compute the upper bound on the reconstruction SNR that can be achieved for a given compression level. From Eq 21 and *α*_SNR_ < 1 we get

$$\begin{array}{c} \log\text{SNR}<\frac{1}{ef}-\log\left(f^{-1}-1\right), \end{array}$$ (22)

which only depends on signal sparsity. For example, if *f* = 0.1, then SNR < 4.4, and the reconstructed signal will not be accurate.

### Extension to continuous variables and other activation functions

Although our analysis above is based on a binary signal / measurement matrix / glomeruli activity and threshold activation function, our results can be extended to positive real-valued signal / measurement matrix / glomeruli activity and any monotonically increasing activation function. Consider the case where the signal *s*^0^ and the element of measurement matrix *A*_*ij*_ could take any positive value rather than just 0 and 1. Denoting *x*^*l*^ = *As*^0^, and letting the activation function *g* be any monotonically increasing function, the output at the glomerulus stage can be written as $x_{i}=g\left(x_{i}^{l}\right)$. Now, signal reconstruction can proceed based on the evaluation of a minimum function (rather than the logical AND function that was used in the case of binary inputs and binary measurement matrices). Indeed, when the *i*th component of the reconstructed signal ${\hat{s}}_{i}$ is computed as the smallest value {*g*^−1^(*x*_*j*_)/*A*_*ji*_} across the set of its inputs (i.e. where *A*_*ji*_ ≠ 0), then our analysis remains valid. The only modification is that now the distribution of the signal and the measurement matrix elements are both required to compute the noise magnitude. This procedure ensures that the recovered components are still recovered exactly, while corrupted components are still corrupted. As a practical aside, we note that the minimum function can be implemented by short-term synaptic plasticity, see S1 Text and S1 and S2 Figs.

## Supporting Information

S1 Text Neural implementation of min function using short-term plasticity. We show by simulation that the output firing rate of a Leaky Integrate and Fire neuron could be well approximated by its minimal input firing rate when synaptic weight is controlled by Short-Term Plasticity.
(PDF)

S1 Fig Simulation results of S1 Text. (EPS)

S2 Fig Simulation results of S1 Text. (EPS)
