# Supplementary material for: A Robust Feedforward Model of the Olfactory System
Source: PLoS Comput Biol. 2016 Apr 11;12(4):e1004850. doi: 10.1371/journal.pcbi.1004850 (PMC4827830; doi:10.1371/journal.pcbi.1004850)
Supplement: S1 Text — We show by simulation that the output firing rate of a Leaky Integrate and Fire neuron could be well approximated by its minimal input firing rate when synaptic weight is controlled by Short-Term Plasticity. (PDF) [file pcbi.1004850.s001.pdf]

S1 Text. Neural Implementation of Minimum Function Using Short-Term Plasticity

The following simulation shows that the Minimum function (output firing rate equals the smallest input firing rate) can be implemented in neural circuits with Short-Term Plasticity (STP).

We use conductance-based Leaky-Integrate-and-Fire (LIF) neuron model with exponentially decaying post-synaptic conductance. The dynamics of the system are given by [1]

$$\tau_m \frac{dv(t)}{dt} = v_{\text{rest}} - v(t) + g_{\text{ex}}(t)[v_{\text{rev}} - v(t)] + I(t)R \tag{1}$$

and

$$\tau_{\text{ex}} \frac{dg_{\text{ex}}(t)}{dt} = -g_{\text{ex}}(t) + w(t) g_{\text{peak}} \delta(t - t_{\text{spk}}), \tag{2}$$

where t_{spk} is the time when the input spike is received. Inhibitory postsynaptic potential is omitted since we only consider excitatory neurons. When $v > v_{\text{thres}}$, the neuron fires a spike and v is reset to v_{reset}. We use the following parameters: $v_{\text{thres}} = -50$ mV, $v_{\text{rest}} = v_{\text{reset}} = -65$ mV, $v_{\text{rev}} = 0$, $\tau_m = 15$ ms, $\tau_{\text{ex}} = 5$ ms, $g_{\text{peak}} = 0.015$, $I = 0$.

Synaptic weight $w(t)$ is described by Short-Term Plasticity. The dynamics are given by [2]

$$w(t) = r(t)u(t)W, \tag{3}$$

$$u(t_{n+1}) = (1 - U)u(t_n) \exp\left(\frac{t_n - t_{n+1}}{\tau_{\text{facil}}}\right) + U, \tag{4}$$

and

$$r(t_{n+1}) = r(t_n)[1 - u(t_{n+1})] \exp\left(\frac{t_n - t_{n+1}}{\tau_{\text{rec}}}\right) + 1 - \exp\left(\frac{t_n - t_{n+1}}{\tau_{\text{rec}}}\right), \tag{5}$$

where t_n is the time of the n-th spike received, $U = 0.6$, $\tau_{\text{facil}} = 5$ ms, $\tau_{\text{rec}} = 100$ ms and W is the synaptic strength.

In the simulation, a LIF neuron receives 6 input spike trains with 6 STP synapses. Each spike train is generated with a fixed firing rate. A Gaussian noise with $\sigma = 1$ ms is added to each spike. The smallest input firing rate $f_{\min}$ is chosen uniformly from 0 to 100 Hz. The other 5 input firing rates are then chosen uniformly from $f_{\min}$ to 200 Hz. Initially $v = v_{\text{rest}}$, $g_{\text{ex}} = 0$, and for each synapse $u = 0$, $r = 1$. Each simulation runs for 2 s, with 1000 trials for each parameter. The results are shown in S1 Fig and S2 Fig.

As shown in S1 Fig, the neuronal output firing rate can be well approximated by its minimal input firing rate when the minimal input firing rate is not high, while the median/mean/max of its input firing rates fail to do that due to the large variance in y-axis. In S2 Fig, we compare the results for different synaptic strength W. As we can see, such approximation is valid for a range of W. Larger W leads to better approximation when the minimal firing rate is high.

References

[1] Burkitt AN. A review of the integrate-and-fire neuron model: I. Homogeneous synaptic input. Biological cybernetics. 2006;95(1):1–19.

[2] Markram H, Wang Y, Tsodyks M. Differential signaling via the same axon of neocortical pyramidal neurons. Proceedings of the National Academy of Sciences. 1998;95(9):5323–5328.
